# Dexmedetomidine and Chloral Hydrate Sedation on ABR Indices in Children

**DOI:** 10.3390/audiolres16040118

**Published:** 2026-08-13

**Authors:** Lan Wang, Qiong Li, Bin Zhang, Shaohan Wang, Shanshan Tian, Yu Han, Wenbin Wang, Zhaomin Fan, Haibo Wang, Yu Ai

**Affiliations:** 1 Department of Otolaryngology, Head and Neck Surgery, Shandong Provincial ENT Hospital, Shandong University, Jinan 250022, China; 201613196@mail.sdu.edu.cn (L.W.); 2019091002@bzmc.edu.cn (Q.L.); shanshantian1@outlook.com (S.T.); yuhan201926@outlook.com (Y.H.); wenbinwang1997@outlook.com (W.W.); fanent@outlook.com (Z.F.); whboto11@email.sdu.edu.cn (H.W.); 2 Clinical Audiology Center, Shandong Second Provincial General Hospital, Jinan 250022, China; 3 Department of Otorhinolaryngology, Qilu Hospital of Shandong University, Jinan 250012, China; zhangbinebyhk@qiluhospital.cn; 4 National Health Commission (NHC) Key Laboratory of Otorhinolaryngology, Shandong University, Jinan 250012, China; 5 School of Integrative Biology, University of Illinois at Urbana-Champaign, Urbana, IL 61801, USA; shaohan3@illinois.edu

**Keywords:** dexmedetomidine, chloral hydrate, auditory brainstem response, children

## Abstract

**Highlights:**

**What are the main findings?**

- With either dexmedetomidine (DEX) or chloral hydrate (CH) sedation, the indices of auditory brainstem responses (ABRs) presented excellent initial test and retest repeatability.
- There was no significant correlation between the average duration of sleep deprivation and the average onset time of sedation in group DEX, whereas a significant negative correlation was observed in group CH.

**What are the implications of the main findings?**

- Regardless of whether the DEX or CH sedative was used, ABR measures demonstrated high test–retest reliability in pediatric patients.
- DEX can be used as a sedative before ABR testing in a pediatric population.

**Abstract:**

**Background/Objectives**: The study was aiming to determine the test–retest repeatability of DEX or CH as single sedative agents on ABR indices by analyzing data from initial test and retest. We also preliminarily explored the correlation between sleep deprivation and onset time of sedation in group DEX and group CH, respectively. **Methods**: We collected data from two groups of children sedated with either DEX (*n* = 20, 40 ears) or CH (*n* = 20, 40 ears). Paired- samples *t*-tests and Bland–Altman plots were used to evaluate repeatability between the initial test and retest. Pearson correlation analysis was used to measure the relationship between the average onset time of sedation and the average duration of sleep deprivation in both groups. **Results**: Within each sedative group, no significant differences were observed in ABR indices between the initial test and retest. Additionally, in group DEX, the mean onset time was 12.15 ± 4.40 min and the mean sleep deprivation duration was 442.80 ± 85.91 min, with no correlation between them (r = 0.01, *p* = 0.96). In group CH, the mean onset time was 27.55 ± 15.70 min and the mean sleep deprivation duration was 455.10 ± 90.94 min, showing a significant negative correlation (r = −0.97, *p* = 0.00). **Conclusions**: ABR indices exhibited high repeatability when DEX and CH were used as a sedative in pediatric ABR examinations. The choice of sedative agent in clinical practice should be individualized, taking into account institutional protocols and clinical requirements.

## 1. Introduction

Auditory brainstem responses (ABRs) [1,2], also known as brainstem auditory evoked potentials (BAEPs), are a type of auditory evoked potential elicited by acoustic stimuli and generated from the inner ear, auditory nerve, and auditory brainstem. It is currently popular and useful in estimating auditory sensitivity, monitoring intraoperative patients, localizing lesions, and screening newborn hearing because of its objectivity and non-invasive properties [3]. It was widely considered as the gold standard for assessing hearing loss in pediatric patients under 2 years old [4]. However, muscular activities could interfere with ABR recordings [5]. To obtain reliable results, infants or children were required to be asleep during the recording of ABRs. Furthermore, children under 8 years old had difficulties in falling asleep naturally [6]. In such cases, the appropriate use of sedatives can facilitate ABR testing effectively.

Chloral hydrate (CH) was a non-opiate, non-benzodiazepine sedative–hypnotic drug, which was a useful pediatric sedative first reported by Carabelle in 1961 [7]. The safety of CH for pediatric sedation remained controversial. Some studies had reported that CH was a relatively safe and effective sedative, while others had argued that it might cause profound respiratory depression, resulting in death or severe neurologic injury even at recommended doses [8]. Owing to its adverse effects and the risk of potential carcinogenic effects (observed in male B6C3F1 mice) [9,10], CH had been banned in some countries. Notwithstanding these adverse effects, CH continued to be utilized for pediatric sedation due to clinician familiarity with this sedative in some other countries. Meanwhile, safer pharmacological alternatives for pediatric sedation had been found [11]. Among these alternatives, dexmedetomidine (DEX), a comparatively emerging sedative, was recognized as an anxiolytic and a sedative that acted through activation of the alpha 2-adrenoreceptor agonists located in the central nervous system [12]. DEX had been increasingly applied to procedural sedation in children because of its reliable sedative properties and safety [13,14]. There were some studies had compared sedation success rate, onset time, adverse reactions and other parameters between DEX and CH in pediatric populations [8,13,14]. However, few studies had described the wave morphology of ABRs after sedation.

Therefore, this study aimed to examine the effect of sedatives (DEX or CH) on ABR indices (latencies of waves I, III, and V; amplitudes of waves I and V; interpeak latencies I–III and I–V; and the wave V threshold) by analyzing data from the initial test and retest, and preliminarily explored the correlation between sleep deprivation and onset time of sedation using DEX or CH as a single sedative.

## 2. Materials and Methods

### 2.1. Participants

**Figure 1:**
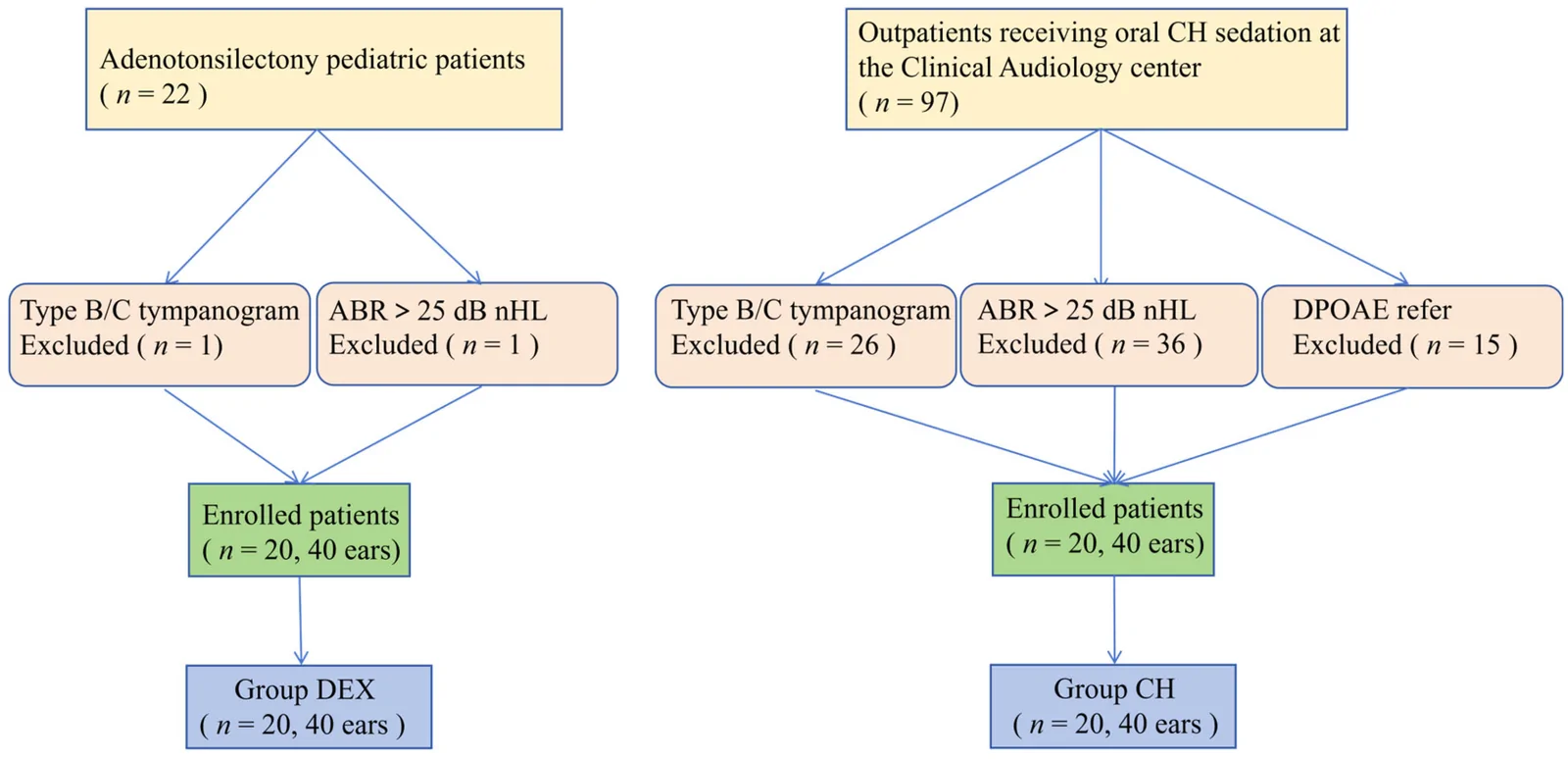
Flow diagram of group DEX and group CH.

Since 2018, at Shandong Provincial ENT Hospital (Jinan, China), pediatric patients undergoing adenotonsillectomy received audiological assessments and DEX sedation via intravenous infusion for preoperative sedation. We screened medical records of 22 adenotonsillectomy patients’ and 97 outpatients with CH- sedated children in the Clinical Audiology Center. Clinical records, audiological testing results, and sedative records were retrospectively reviewed. We included patients with the following criteria: (1) otoscopy revealed clean external auditory canal and healthy tympanic membrane; (2) ABR was within the normal range (≤25 dB nHL, click stimuli); (3) all participants passed behavioral audiometry (BA) in both ears; (4) the 226 Hz tympanogram was Type A; (5) all participants were required to pass distortion product otoacoustic emission (DPOAE) in both ears. The exclusion criteria were as follows: (1) poor compliance or cooperation; (2) absence of parental or legal guardian consent; (3) the participant’s voluntary refusal to engage in the study. Finally, 20 subjects (40 ears) who were systematically assigned to group DEX, as well as 20 age- and gender-matched controls (group CH), were recruited. The flow diagram is presented in Figure 1.

This study was approved by the Ethics Review Committee of Shandong Provincial ENT Hospital (China approval number: XYK-20190310).

### 2.2. Sedation Method

Pre-sedation assessments were conducted by an anesthesiologist and included the following criteria: (1) no upper respiratory tract infection or fever; (2) no history of respiratory arrest, either currently or within the preceding 3 months; (3) no bradycardia, severe congenital heart disease, or other significant cardiovascular issues; (4) fasting for 3 h and water deprivation for 2 h before sedation. The sedation procedure was performed by an experienced anesthesiologist who evaluated the depth of sedation. DEX was diluted with physiological saline and administered intravenously with an infusion pump (0.6 μg/(kg·h)). CH was administered as a 10% solution at a dose of 50 mg/kg orally for patients, with a maximum total dose of 1 g in 24 h.

Definitions: (1) Successful sedation: the Ramsay score was higher than 4 and the child could receive the electrophysiological examination. (2) Sedation onset time: the time from the administration of the sedative to the successful sedation. (3) Sedation failure: the Ramsay score was lower than 5 in 90 min after sedative administration, the child woke up during ABR recording, or the tests could not be performed due to body movements [15]. (4) Sleep deprivation duration: the period of continuous wakefulness from the patient’s last awakening until the administration of the sedative.

The adverse effects of two sedatives were recorded in this study; adverse effects were classified as severe or minor. The severe adverse effects associated with DEX were: (1) emergency airway intervention, (2) severe arrhythmia, (3) respiratory and cardiac arrest, etc. The minor adverse effects were as follows: (1) bradycardia, (2) a significant oxygen saturation decrease, (3) upper respiratory tract obstruction, etc. [16]. For CH, the severe adverse effects were: (1) respiratory arrest, (2) severe arrhythmia, (3) death, etc. The minor adverse effects were as follows: (1) vomiting, (2) tachypnea, (3) rash, etc. [17,18,19]. Children should be accompanied by at least one parent. They were monitored with finger pulse oximetry, and an emergency cart was ensured for all participants during sedation.

### 2.3. Apparatus and Procedure

The audiological records included tympanogram (Tympstar Pro, Grason-Stadler, MN, USA), BA (Audiostar Pro, Grason-Stadler, MN, USA), DPOAE (Bio-logic Scout Sport, Natus, IL, USA), and ABR (Bio-logic Navigator Pro, Natus, USA). The ABR recording parameters were as follows: (1) The impedance was <5 KΩ; (2) the reference electrodes were positioned on the ipsilateral mastoid of the subject, while the recording electrode was placed at the midpoint of the head (Cz), and the ground electrode was situated on the forehead (Fpz); (3) the stimulus was sent to the ear through an insert earphones (air conduction), and the test ear was stimulated by 80 dB nHL click sound at rates of 27.5 stimuli per sec (1024 sweeps); (4) the ABR threshold was defined as the minimum sound intensity sufficient to elicit a reproducible wave V. During the ABR test, the participants received two recordings each stimulus intensity (these two recordings were the initial test and retest, respectively). All electrophysiological examinations were conducted in a sound- attenuated, electrically shielded room.

### 2.4. Statistical Analysis

All statistical analyses were performed using SPSS (version 19.0, IBM Corp, NY, USA.). Normality of data distribution was first assessed via the Shapiro–Wilk test. For the primary analysis, paired- sample *t*-tests were used to compare the ABR latencies and amplitudes between the initial test and retest measurements within group DEX and group CH, respectively. Bonferroni correction was applied for multiple comparisons, with the adjusted α = 0.005. All reported *p*-values were two-tailed and had been adjusted accordingly.

Secondly, Bland–Altman plots were constructed to evaluate the agreement between initial test and retest recording: the plots were bivariate scatter diagrams. The X-axis represented the mean of the initial test and retest, and the Y-axis showed the difference between them. Good agreement was defined as a mean difference close to zero and the majority of scatter points falling within the 95% limits of agreement.

Finally, Pearson correlation tests were used to assess the correlations between the average onset time of sedation and the average duration of sleep deprivation. Outlier diagnostics were conducted via scatter- plot, box-plot (1.5 × IQR rule), and Z-score standardization (|Z| > 3). Both Pearson and Spearman correlation coefficients were computed. Robustness was assessed using leave-one-out sensitivity analysis and paired bootstrap 95% confidence intervals. *p*-value < 0.05 was considered statistically significant.

## 3. Results

### 3.1. Demographic Information

Demographic characteristics are presented in Table 1 for both groups. Group DEX comprised 11 males and 9 females with a mean age of 5.48 ± 1.52 years; group CH included 12 males and 8 females, with a mean age of 5.28 ± 1.34 years. There were no significant differences in gender and age between the two groups.

### 3.2. Comparison of Within-Group Repeatability of ABR Latencies and Amplitudes Between the Initial Test and Retest

Table 2 showed the latencies and amplitudes of ABR waves recorded in the initial test and retest with Bonferroni correction applied to maintain statistical rigor. The mean differences between the two measurements ranged from −0.02 to +0.01 across indices. Paired *t*-tests yielded *p*- values ranging from 0.11 to 1.00, and Wilcoxon signed-rank tests confirmed these findings with consistent results (all *p* > 0.05), supporting the robustness of parametric tests against skewed distributions at *n* = 40. In both groups, the latencies and amplitudes were not significantly different between the initial test and retest (*p* > 0.05).

**Figure 2:**
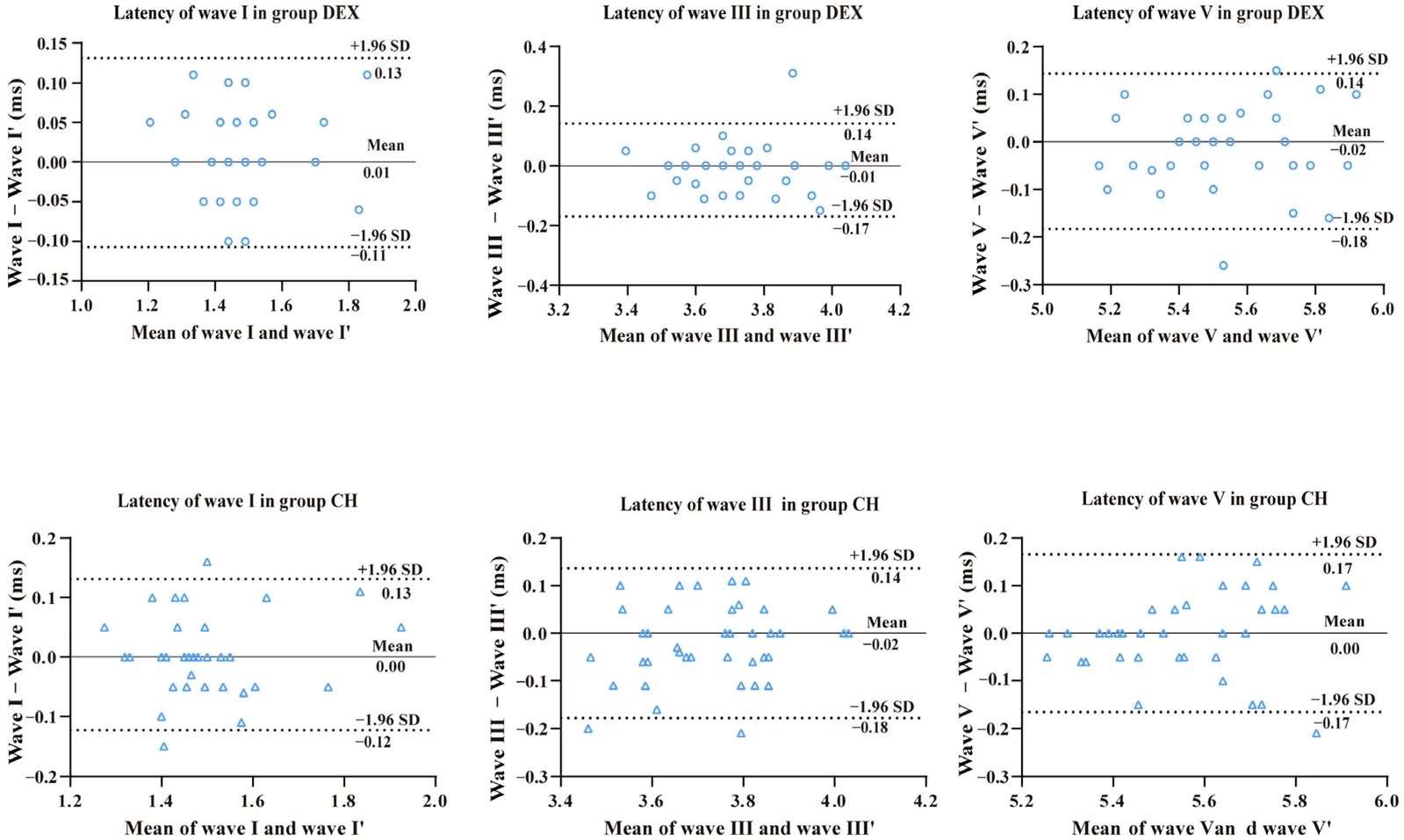
Bland–Altman plots for latencies as a function of DEX and CH. DEX: DEX sedation group; CH: CH sedation group. The X-axis represents the average of the initial test and retest latency measurements, while the Y-axis represents the difference between them. Solid lines represent average mean test difference. Dashed lines represent SD (i.e., limits of agreement).

**Figure 3:**
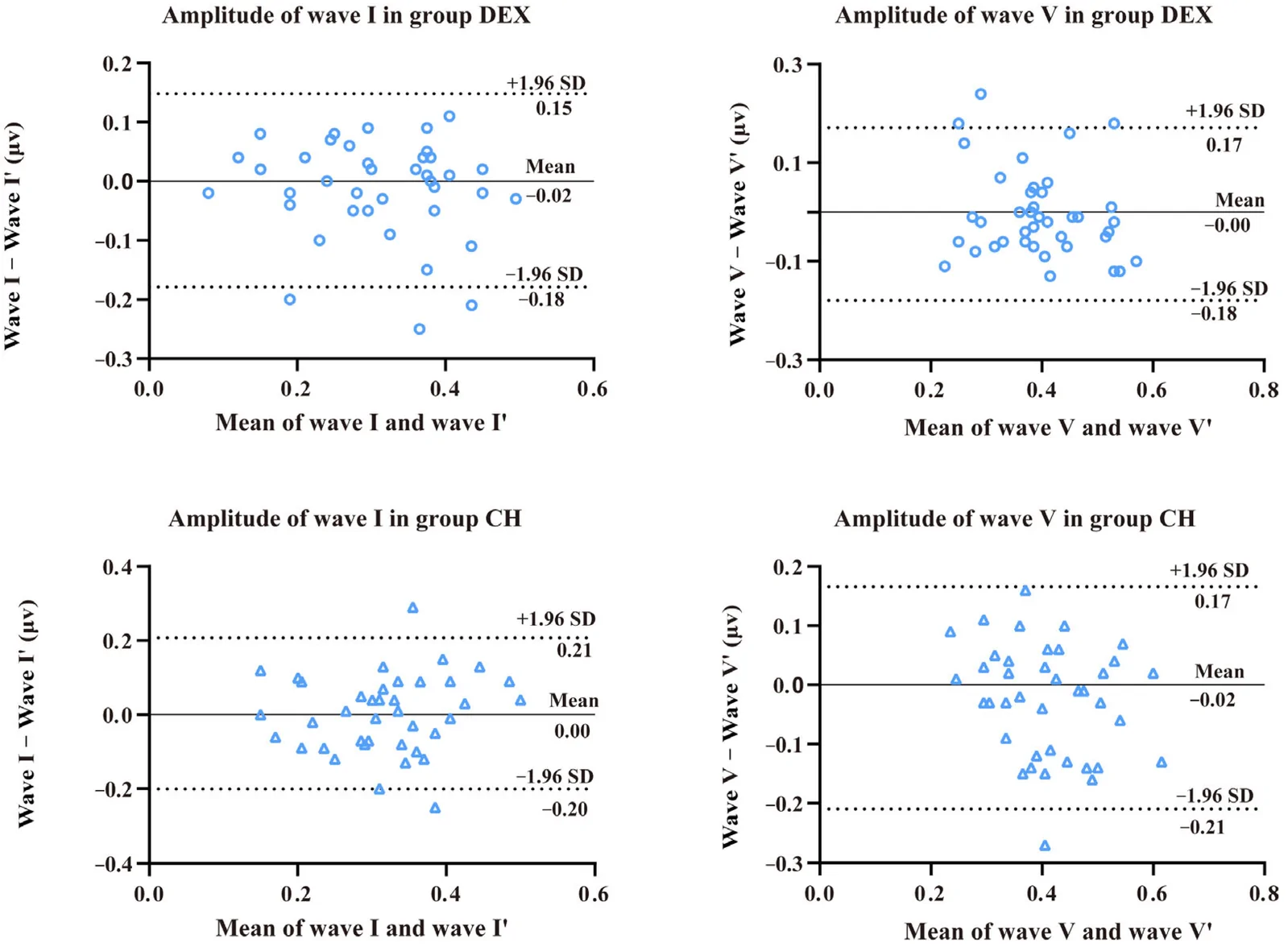
Bland–Altman plots of amplitudes as a function of DEX and CH. DEX: DEX sedation group; CH: CH sedation group. The X- and Y-axis represent the average of the initial test and retest amplitude measurements and the difference between them, respectively. Solid lines represent average mean test difference. Dashed lines represent SD (i.e., limits of agreement).

Bland–Altman analysis showed good agreement between the latencies of the initial test and retest. In group DEX, the mean differences in latencies for waves I, III, and V between the initial test and retest records were 0.01, −0.01, and −0.02, respectively (Figure 2). As for group CH, the mean differences in latencies for waves I, III, and V between the initial test and retest records were 0.00, −0.02, and 0.00 (Figure 2). In group DEX, the mean amplitude differences for waves I and V between the initial test and retest records were −0.02 and −0.00, respectively (Figure 3). Meanwhile, in group CH, the mean amplitude differences of waves I and V between the initial test and retest records were 0.00 and −0.02, respectively (Figure 3). The mean differences were found close to zero, with the vast majority of data points falling within the range of 1.96 standard deviations above and below the mean differences.

### 3.3. ABR Parameters in Groups DEX and CH

Table 3 presented the ABR indices for groups DEX and CH, respectively. For each parameter, the mean values from the initial and repeated ABR tests were averaged across all 40 ears. ABR latencies for waves I, III, and V were 1.48 ± 0.13, 3.72 ± 0.15, and 5.54 ± 0.20 ms in group DEX, and 1.49 ± 0.13, 3.73 ± 0.15, and 5.54 ± 0.18 ms in group CH, respectively. Amplitudes for waves I and V were 0.31 ± 0.11 and 0.40 ± 0.10 μV in group DEX, and 0.32 ± 0.10 and 0.41 ± 0.10 μV in group CH. Interpeak latencies for I–III and I–V intervals were 2.25 ± 0.14 and 4.06 ± 0.19 ms in group DEX, and 2.24 ± 0.14 and 4.06 ± 0.18 ms in group CH. ABR thresholds were 17.50 ± 6.50 dB nHL in group DEX and 18.75 ± 5.40 dB nHL in group CH.

### 3.4. Outcomes with Two Groups—Clinical Measures

As shown in Table 4, in group DEX, the average onset time of sedation was 12.15 ± 4.40 min and the average duration of sleep deprivation was 442.80 ± 85.91 min. In group CH, the average onset time of sedation was 27.55 ± 15.70 min and the average duration of sleep deprivation was 455.10 ± 90.94 min. The sedation success rate was 100% in both groups. No adverse effect was observed in group DEX, while 10% (2/20) of participants in group CH presented vomiting, which were mild and self-limiting.

**Figure 4:**
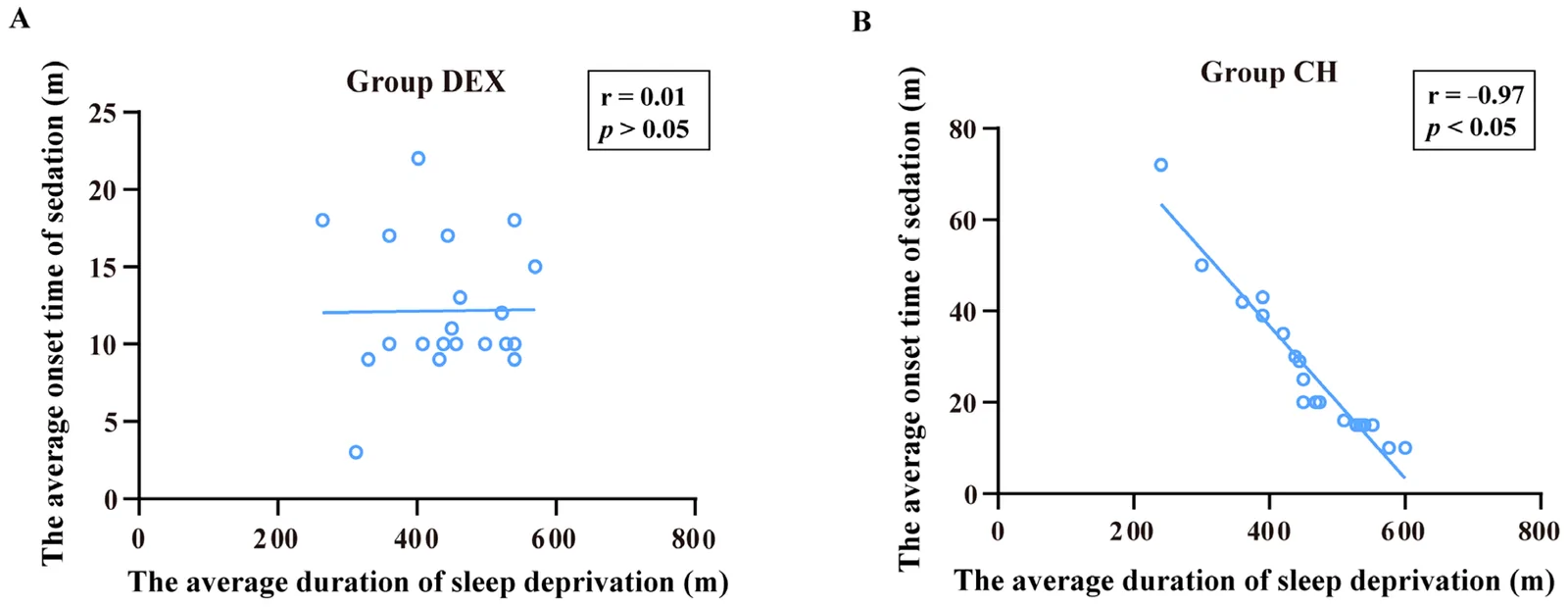
(**A**) The average onset time of sedation vs. the average duration of sleep deprivation in group DEX; (**B**) The average onset time of sedation vs. the average duration of sleep deprivation in group DEX. DEX: DEX sedation group; CH: CH sedation group.

Figure 4A,B showed the relationship between the average onset time of sedation and the average duration of sleep deprivation in both groups. In group DEX, we found no significant correlation between the average onset time of sedation and the average duration of sleep deprivation (r = 0.01, *p* = 0.96). In contrast, a significant negative correlation was found between the two variables (r = −0.97, *p* = 0.00) in group CH.

## 4. Discussion

The repeatability of ABR waveforms served as a fundamental prerequisite for reliability or agreement of inter-/intra-operator [20,21,22]. Moreover, a better repeatability of ABR could make the diagnostic results more reliable. Although previous studies had established the equivalence of various sedatives in ABR testing, the repeatability using different sedatives had not been evaluated [23,24]. In the current study, the initial test and retest reliability of ABR with two different sedatives was examined in two ways. On the one hand, the paired- samples *t*-test was determined to examine the consistency between initial test and retest of the ABR indices for both sedatives. It revealed no statistically significant differences in latencies or amplitudes between initial test and retest, whether by using DEX or CH for sedation (*p* > 0.05) (Table 2). On the other hand, because of the limitations of relying solely on statistical significance to assess reliability, Bland–Altman plots were constructed to examine test–retest reliability between the initial test and retest measurements of ABR indices under each sedative condition [25]. In our study, Bland–Altman analysis revealed minimal mean differences in latencies and amplitudes between initial test and retest recordings, with most data points falling within the 95% limits of agreement, which was consistent with the expectation that mean test differences should be zero and 95% of differences be within ± 1.96 SD. This was apparent in all plots (Figure 2 and Figure 3). Considering all analyses, we could conclude that ABR measures were reliable in children following the initial test and retest measurements, regardless of whether DEX or CH sedation was used.

In the present study, all ABR parameters (including absolute latencies, amplitudes, interpeak latencies, and thresholds) obtained under DEX sedation showed a similar trend to those obtained under CH sedation (Table 3). Nevertheless, since the two groups were not derived from a parallel-controlled design, formal intergroup statistical analysis was not performed. Therefore, the observed “similarity” merely reflected the numerical proximity of the parameter values, and the possibility of potential differences could not be statistically excluded. Notably, owing to lack of natural-sleep control group, it was not sufficient to conclude that DEX or CH had no effects on ABR or neural conduction. In addition, it should be noted that the present study only enrolled normal-hearing children. Whether these commonly used sedatives affect ABR thresholds and latencies in children with pre-existing hearing impairment warrants further investigation.

In our research, the average onset time of sedation in group DEX was 12.15 ± 4.40 min, which was shorter than that in group CH (27.55 ± 15.70 min) (Table 4). It corresponded to previous reports [11,13], whereas the average onset time for both sedatives in the current study was shorter than reported by Jason et al. The relative discrepancy of the aforementioned findings might be attributable to different factors, including different demographic and clinical characteristics, relatively small sample sizes, pre-procedure sleep deprivation, routes of sedative agents, etc. Notably, intravenous administration of DEX, as used in the present study, bypasses the absorption phase required for intranasal or oral routes, thus achieving a more rapid onset. Furthermore, most subjects of group DEX have upper-airway hypertrophy or obstructive sleep apnoea, which might lead them to falling asleep faster.

Although previous studies adopted intranasal administration [13,26] and the present study used intravenous administration, all achieved a 100% sedation success rate, which implied that the efficacy of DEX was likely independent of the route of administration. Conversely, CH exhibited various sedation success rates: 90.5% by Jason et al. [13], 95.9% across 697 patients by Dianne G et al. [19], and 100% in infants under 6 months versus 72% in older children by Eirini et al. [17]. Previous studies had generally reported higher sedation success rate with DEX than with CH [8,13]. A 100% success rate was achieved with both sedatives in this investigation (Table 4). The outcome was potentially ascribed to the following factors: (1) stricter enforcement of sleep deprivation protocols, (2) differences in subjects age distribution, and (3) the relatively limited sample size.

In this study, we recorded adverse effects related to the use of DEX or CH. Significantly, no adverse (include respiratory adverse effects or gastrointestinal side effects) effect was observed in group DEX (0% incidence), with oxygen saturation levels maintained above 92% in all subjects (Table 4). This might be owing to the rigorous, continuous monitoring inherent to an inpatient surgical setting. In addition, only 20 subjects were enrolled in group DEX. Although, a small sample size would preclude definitive conclusions about safety, the findings of the present study were consistent with recent evidence from Nie et al., in which no significant difference in respiratory adverse effects were demonstrated among 859 pediatric DEX sedation versus controls (OR = 0.70; 95% CI 0.48–1.02) [27]. Similarly, Li et al. and Yuen et al. stated that rare occurrences of respiratory depression were documented during pediatric DEX administration [28,29]. These large-scale studies, with substantially larger sample sizes than the present investigation, further corroborating the safety profile of DEX observed in our study. Furthermore, 10% (2/20) participants in group CH presented vomiting, which was mild and self-limiting (Table 4). The gastrointestinal side effects associated with CH sedation were in agreement with those evidenced in previous studies [30]. It was critical to continuously monitor vital signs to ensure clinical safety, even if adverse effects were uncommon in the previous investigation and the present study.

In pediatric populations, the efficacy of DEX-based sedation had been increasingly recognized. A recent meta-analysis of intranasal DEX in children undergoing tonsillectomy and/or adenoidectomy reported significant reductions in emergence agitation and delirium [31], underscoring its clinical utility in this demographic. However, that analysis also highlighted limitations inherent in the current evidence base, including small sample sizes, substantial heterogeneity across dosing regimens, and the predominance of single region studies, which collectively temper broad generalizability [31]. Similarly, another meta-analysis comparing DEX with remimazolam for sedation during regional anesthesia in adult surgical patients demonstrated favorable safety and efficacy profiles for DEX [32], though direct extrapolation to pediatric procedural sedation warrants caution due to age-related pharmacokinetic differences.

Regardless of the increasing use of DEX in pediatric populations, limited research had been conducted on the implementation of sleep deprivation prior to DEX sedation. In the current study, we found that there was no correlation between the mean onset time of sedation and the mean duration of sleep deprivation (Figure 4A). This finding suggested that the duration of sleep deprivation did not affect the onset time of sedation of DEX. It was based on this finding that DEX acted specifically on the central ɑ2-adrenoreceptor agonist in the locus coeruleus, independent of homeostatic sleep drive [33,34]. A mechanism corroborated by recent animal studies on DEX sedation in feline models [35,36]. Individual variability in receptor sensitivity or drug metabolism might also mask subtle effects [37,38]. It implied that DEX sedation might be independent from sleep homeostasis. This property was particularly valuable in pediatric settings, where enforcing controlled sleep deprivation was often impractical and poorly tolerated. However, due to the small sample size, we were unable to perform a comparison of sedation onset time between patients with and without sleep deprivation. Therefore, we could not conclude whether sleep deprivation should be necessary for DEX sedation.

In addition, we observed a significant negative correlation between the two variables in group CH (r = −0.97; *p* < 0.05) (Figure 4B). This finding suggested that longer mean sleep- deprivation time was associated with shorter mean sedation- onset time. So far, the clinical utility of sleep deprivation prior to CH sedation remained debatable. Some scholars had contended that the hypothesis linking sleep deprivation to enhanced efficacy of CH lacked empirical validation [39,40]. For instance, Cui et al. (2021) found no effect of sleep deprivation on the success rate of chloral hydrate sedation in a large- propensity score-matched cohort (n = 7789) [37]. Our finding did not align with that of Cui et al. It could be attributable to sample size and methodological differences. Cui et al. employed a binary outcome (sedation success vs. failure), whereas the present study measured continuous onset time; supplemental dose was permitted in their sedation regimen; furthermore, most of procedures were relatively brief and quick in their study, while ABR testing requires sustained deep sleep, placing substantially greater demands on homeostatic sleep pressure. Other studies had reported that combining sleep deprivation with oral 10% CH significantly shortened onset time of sedation before ABR testing in children [41]. Our observation in group CH was consistent with the latter view. Sleep deprivation might act as an adjuvant to potentiate the sedative effects of CH, possibly via the additive interaction between homeostatic sleep pressure and central CH inhibition, leading to a faster sedative response [42]. Nevertheless, these results should be considered preliminary.

## 5. Limitations

Despite its clinical relevance, this study had several limitations. First, the sample size was relatively small, which might have limited the statistical power and generalizability of our findings. Second, the allocation of DEX versus CH was determined by clinical pathways rather than randomization. Specifically, group DEX comprised surgical patients undergoing adenotonsillectomy, while group CH consisted of outpatients. These two populations differed in clinical setting, baseline health status, and comorbidities (e.g., OSA in group DEX), which inevitably introduced selection bias. Although both groups had normal hearing, systemic conditions unique to each population might have influenced sedation. Therefore, our study should not be viewed as a strictly parallel comparative trial; rather, it described the safety profiles of two sedation protocols in distinct clinical scenarios. Caution should be warranted when generalizing our conclusions. Future studies with larger sample sizes should employ a randomized controlled design comparing consistent routes of administration (e.g., intranasal DEX vs. oral CH) in similar clinical settings.

## 6. Conclusions

Both DEX and CH presented outstanding initial test and retest repeatability on ABR indices when applied for pediatric ABR sedation. Nevertheless, the present study was not designed to directly compare the two sedatives, precluding any determination of superiority. In clinical practice, the selection of an appropriate sedative agent should be tailored based on individual patient characteristics, institutional preferences, and specific clinical requirements. Continuous monitoring should be an integral component throughout the entire sedation.

## Figures and Tables

**Table 1:** Demographic characteristics ($\bar{x}\pm s$).

	Number of People (Male/Female)	*p*	Age (2–8 Years Old)	*p*
DEX	20 (11/9)	1.00	5.48 ± 1.52	0.67
CH	20 (12/8)		5.28 ± 1.34	

Note: DEX: DEX sedation group, CH: CH sedation group.

**Table 2:** Latencies and amplitudes of ABR waves in the initial test and retest ($\bar{x}\pm s$).

	I	I’	*p*	III	III’	*p*	V	V’	*p*
DEX (Wave latency ms)	1.48 ± 0.13	1.47 ± 0.13	0.22	3.71 ± 0.15	3.73 ± 0.15	0.26	5.53 ± 0.20	5.55 ± 0.20	0.14
CH (Wave latency ms)	1.49 ± 0.14	1.48 ± 0.13	0.68	3.72 ± 0.16	3.74 ± 0.14	0.11	5.54 ± 0.18	5.54 ± 0.17	1.00
DEX (amplitude μV)	0.30 ± 0.10	0.32 ± 0.11	0.25	-	-	-	0.39 ± 0.09	0.40 ± 0.11	0.78
CH (amplitude μV)	0.32 ± 0.10	0.31 ± 0.09	0.85	-	-	-	0.40 ± 0.09	0.42 ± 0.11	0.16

Note: DEX: DEX sedation group; CH: CH sedation group; I, III, V: the initial test of waves I, III, and V; I’, III’, V’: all were the retest of waves I, III, and V.

**Table 3:** ABR latencies, amplitudes, interpeak latencies (80 dB nHL click), and thresholds in group DEX and group CH ($\bar{x}\pm s$).

	DEX	CH
	Wave Latency (ms)	Wave Latency (ms)
I	1.48 ± 0.13	1.49 ± 0.13
III	3.72 ± 0.15	3.73 ± 0.15
V	5.54 ± 0.20	5.54 ± 0.18
	Amplitude (μV)	Amplitude (μV)
I	0.31 ± 0.11	0.32 ± 0.10
V	0.40 ± 0.10	0.41 ± 0.10
	Interpeak Latency (ms)	Interpeak Latency (ms)
I–III	2.25 ± 0.14	2.24 ± 0.14
I–V	4.06 ± 0.19	4.06 ± 0.18
	Threshold (dB nHL)	Threshold (dB nHL)
	17.50 ± 6.50	18.75 ± 5.40

Note: DEX: DEX sedation group; CH: CH sedation group.

**Table 4:** Relevant information of sedation in both groups ($\bar{x}\pm s$).

	The Average Onset Time of Sedation (Minutes)	The Average Duration of Sleep Deprivation (Minutes)	Sedation Success Rates	Incidence of Adverse Effects
DEX	12.15 ± 4.40	442.80 ± 85.91	100%	0% (0/20)
CH	27.55 ± 15.70	455.10 ± 90.94	100%	10% (2/20)

Note: DEX: DEX sedation group; CH: CH sedation group.

## Data Availability

The datasets used and/or analyzed during the current study are available from the corresponding author on reasonable request.
